# Catecholamine concentration as a predictor of mortality in emergency surgical patients

**DOI:** 10.1186/s12245-024-00676-4

**Published:** 2024-07-18

**Authors:** João Isuk Suh, Daiane Leite da Roza, Filipe Matheus Cadamuro, Luiz Marcelo Sá Malbouisson, Talita Rojas Sanches, Lúcia Andrade

**Affiliations:** 1 Nephrology Division, Hospital das Clínicas, University of São Paulo School of Medicine, São Paulo, Brazil; 2 Department of Epidemiology, School of Public Health, University of São Paulo, São Paulo, Brazil; 3 Trauma Intensive Care Unit, Hospital das Clínicas, University of São Paulo School of Medicine, São Paulo, Brazil; 4 Division of Nephrology, University of São Paulo School of Medicine, Av. Dr. Arnaldo, 455, 3º andar, sala 3310, São Paulo, SP CEP 01246-903 Brazil

**Keywords:** Norepinephrine, Epinephrine, SOFA, SAPS, ICU outcome, Vasopressors, Mortality

## Abstract

**Background:**

Trauma and emergency surgery are major causes of morbidity and mortality. The objective of this study was to determine whether serum levels of epinephrine and norepinephrine are associated with aging and mortality.

**Methods:**

This was a prospective observational cohort study conducted in a surgical critical care unit. We included 90 patients who were admitted for postoperative care, because of major trauma, or both. We collected demographic and clinical variables, as well as serum levels of epinephrine and norepinephrine.

**Results:**

For patients in the > 60-year age group, the use of vasoactive drugs was found to be associated with an undetectable epinephrine level (OR [95% CI] = 6.36 [1.12, 36.08]), *p* = 0.05). For the patients with undetectable epinephrine levels, the in-hospital mortality was higher among those with a norepinephrine level ≥ 2006.5 pg/mL (OR [95% CI] = 4.00 [1.27, 12.58]), *p* = 0.03).

**Conclusions:**

There is an association between age and mortality. Undetectable serum epinephrine, which is more common in older patients, could contribute to poor outcomes. The use of epinephrine might improve the clinical prognosis in older surgical patients with shock.

## Background

Trauma is one of the main causes of disability and mortality among adults worldwide, having a major socioeconomic impact and increasing health care costs considerably [1, 2]. Age has been reported to be a robust predictor of morbidity and mortality in critically ill patients, including those with severe trauma [3, 4]. Johansson et al. [5] demonstrated an association between age and mortality in trauma patients. The authors found that plasma norepinephrine levels were higher in older patients. However, they also found that epinephrine release was attenuated in proportion to the increase in the Injury Severity Score in the older patients, who also showed impaired mobilization of platelets and leukocytes, as well as increased consumption of anticoagulants and hyperfibrinolysis, all of which can lead to poor outcomes [6]. Brattström et al. [7] evaluated trauma patients admitted to the intensive care unit (ICU) and found that being ≥ 55 years of age was an independent predictor of multiple organ failure, severe sepsis, and mortality. The importance of age for outcomes in critically ill patients is also illustrated by its inclusion in scores aimed at stratifying risk in such patients, as exemplified by the Acute Physiology and Chronic Health Evaluation score. Hypothalamic–pituitary–adrenal axis activation leads to the secretion of glucocorticoids, whereas activation of the sympathetic nervous system leads to the secretion of catecholamines, together with complex interactions between the immune and nervous systems [8].

The purpose of this study was to determine whether the serum levels of epinephrine and norepinephrine at admission correlate with age and mortality in emergency surgical patients, including trauma and non-trauma patients. We hypothesized that the use of vasopressin or norepinephrine would be associated with serum epinephrine undetectability in patients over 60 years of age, as well as that when the serum level of epinephrine is undetectable, a higher serum norepinephrine level would be associated with a greater risk of death.

## Methods

### Study design

This was a prospective observational cohort study of trauma and emergency surgical patients admitted to the ICU of a quaternary hospital. We report findings related to a cohort of 90 patients recruited in order to measure catecholamine levels at ICU admission.

### Patient selection

The study was carried out in a specialized ICU for victims of major trauma and emergency surgery in the city of São Paulo, Brazil. We included all patients who were at least 18 years of age, although we excluded those who were pregnant. Data were collected from the ICU admission records, and blood samples were collected to measure serum levels of epinephrine and norepinephrine. We collected demographic and clinical data: sex; age; history of disease (diabetes mellitus, chronic obstructive pulmonary disease, hypertension, immunosuppression, congestive heart failure, coronary insufficiency, chronic kidney disease, or cancer); and the Sequential Organ Failure Assessment (SOFA) score, Glasgow Coma Scale score, and Simplified Acute Physiology Score III (SAPS III) at admission. We also collected data related to the use of mechanical ventilation, vasoactive drugs such as norepinephrine (µg/kg per minute), epinephrine (µg/kg per minute), and vasopressin (units per hour), as well as the surgical site (abdomen, brain, chest, or extremity).

### Blood samples

Blood samples were centrifuged, and the serum was stored at − 80 °C. The serum levels of catecholamines (epinephrine and norepinephrine) were measured by high-performance liquid chromatography (pg/nL).

### Statistical analysis

Categorical variables are expressed as absolute and relative frequencies, whereas continuous variables are expressed as means and standard deviations, with ranges, with or without medians and interquartile ranges. In order to analyze the variables that characterize the sample, we performed univariable and multivariable analyses, categorical data analysis, parametric hypothesis tests, and logistic regression (adjusted by the Cook and Atkinson method). Pearson’s linear correlation coefficient was applied to determine whether in-hospital mortality correlated with the SAPS III or SOFA score. We adopted a 95% confidence interval (95% CI), and values of *p* < 0.05 were considered statistically significant.

## Results

### Patients

Of the 90 patients evaluated, 62 (68.9%) were men and only 28 (31.1%) were women. All of the patients underwent emergency surgery, trauma patients accounting for 66.7% (*n* = 60) and non-trauma patients accounting for 33.3% (*n* = 30). A total of 60 patients (66.7%) required mechanical ventilation: 44 (73%) were trauma patients (all of whom required mechanical ventilation less than 24 h before ICU admission), and 16 (27%) were non-trauma patients (two of whom required mechanical ventilation more than 48 h before ICU admission). Table 1 shows the demographic and clinical characteristics of the patients.

**Table 1:** Demographic and clinical characteristics of the patients

Variable	*n*	Min.	Mean	Median	Max.	SD
Age (years)	90	18.0	54.5	54.5	91.0	19.3
SOFA score (D0)	90	0.0	7.2	7.5	18.0	4.1
SOFA score (D1)	90	0.0	5.5	4.0	18.0	4.7
SAPS III score	90	17.0	53.2	53.0	103.0	17.2
Glasgow Coma Scale score	90	3.0	8.3	7.0	15.0	5.2
Body temperature (°C)	90	34.0	36.2	36.1	39.5	0.9
Systolic blood pressure (mmHg)	90	77.0	121.7	120.0	181.0	24.4
Heart rate (bpm)	90	45.0	96.0	98.0	150.0	22.6
Urinary volume (mL/24 h; D1)	85	0.0	1276.6	1200.0	3200.0	819.9
C-reactive protein (mg/L)	77	0.3	112.1	54.9	779.0	145.4
Lactate (mg/dL)	87	4.0	31.6	21.0	193.0	35.8
Leucocyte count (cells/mm^3^)	90	1710.0	15818.4	13940.0	44860.0	8035.1
Platelet count (cells/mm^3^)	90	28000.0	240955.6	244000.0	629000.0	111931.2
pH	90	7.0	7.3	7.3	7.6	0.1
Creatinine (mg/dL; D1)	90	0.5	1.6	1.3	6.2	1.2
Creatinine (mg/dL; D2)	88	0.5	1.7	1.1	6.0	1.3
Bilirubin (mg/dL)	88	0.1	0.9	0.5	6.7	1.2
PaO_2_ (mmHg)	87	0.6	123.3	100.0	367.0	67.5
ICU stay (days)	90	1.0	10.8	7.0	72.0	12.0
Hospital stay (days)	87	1.0	24.3	16.0	128.0	24.5

*Min.* minimum, *Max.* maximum, *SD* standard deviation, *SOFA* Sequential Organ Failure Assessment, *SAPS III* Simplified Acute Physiology Score III, *D0* day 0 (admission), *D1* day 1, *D2*, day 2, *ICU* intensive care unit, *bpm* beats per minute

### Levels of epinephrine and norepinephrine

Table 2 shows that the serum levels of epinephrine and norepinephrine both presented a wide dispersion, with standard deviations approximately 3 and 2 times greater than their respective means. We also observed that the mean levels of epinephrine and norepinephrine were much higher than the corresponding median values and third quartiles, increasing the asymmetry of the distribution for those variables. It is noteworthy that 60 (66.7%) of the 90 patients presented undetectable serum levels of epinephrine, and the corresponding data were therefore omitted for the calculation of descriptive measures. Undetectable serum epinephrine was seen mainly in the older patients who were treated with vasoactive drugs (Table 3).

**Table 2:** Serum levels of epinephrine and norepinephrine at admission

Variable	*n*	Minimum	Quartile 1	Median	Quartile 3	Maximum	Mean	SD
Epinephrine (pg/mL)	30^a^	62.0	337.0	568.5	4890.0	410994.0	26114.4	80141.2
Norepinephrine (pg/mL)	90	119.0	650.0	2685.5	12598.0	120601.0	10960.0	21092.4

^a^Serum epinephrine was undetectable in 60 patients

**Table 3:** Levels of Norepinephrine in patients with undetectable levels of Epinephrine

Variable	*n*	Minimum	Quartile 1	Median	Quartile 3	Maximum	Mean	SD
Norepinephrine (pg/mL)	60	119.0	591.5	2006.5	9176.0	58011.0	6138.2	9648.5

### Epinephrine detection

At admission, 34 patients were not receiving any vasoactive drugs, 46 were receiving norepinephrine, vasopressin, or both and 8 were also receiving epinephrine. None of the 60 patients who presented undetectable serum levels of epinephrine were receiving exogenous epinephrine infusion (Table 4). However, of the patients with the highest serum levels of epinephrine at admission, seven patients were receiving exogenous epinephrine infusion. Table 4 shows the frequency of administration of epinephrine and of vasopressin/norepinephrine, by group (detectable vs. undetectable serum epinephrine). Among the 46 patients who were receiving some combination of norepinephrine and vasopressin, the serum level of epinephrine was undetectable in 36, compared with only 24 of the 36 patients who were receiving no norepinephrine or vasopressin. However, that difference was not significant.

**Table 4:** Frequency distribution of patients, stratified by detectability of serum epinephrine and vasoactive drug administered

	Serum epinephrine		Total	*p*-value*	OR (95% CI)
	Undetectable	Detectable			
Patients receiving epinephrine, *n* (%)					
No	60 (67.7)	22 (24.4)	82 (91.1)	**< 0.01**	**-**
Yes	0 (0.0)	8 (8.9)	8 (8.9)		-
Total	60 (66.7)	30 (33.3)	90 (100.0)		
Patients receiving vasopressin, norepinephrine, or both, *n* (%)					
No	24 (29.3)	12 (14.6)	36 (43.9)	0.31	1.00 (Reference)
Yes	36 (43.9)	10 (12.2)	46 (56.1)		0.55 (0.21; 1.49)
Total	60 (73.2)	22 (26.8)	82 (100.0)		

*OR* odds ratio; *95% CI* 95% confidence interval

*Fisher’s exact test

### Age and serum levels of epinephrine

We stratified the sample by age, evaluating those ≤ 60 years of age in comparison with those > 60 years of age, in order to determine whether age influenced the association between undetectable serum epinephrine and the administration of vasopressin or norepinephrine. We chose the age of 60 years because it is close to the median age of the cohort, resulting in balanced groups for analysis (Table 5). For patients in the > 60-year age group, the use of vasoactive drugs was found to be associated with undetectable levels of epinephrine, (OR [95% CI] = 6.36 [1.12, 36.08]), *p* = 0.05). Another relevant relationship observed was among the serum epinephrine level, the administration of vasoactive drugs, and the surgical site. Of the 90 patients evaluated, 29 (32.2%) underwent brain surgery, 19 (21.1%) underwent thoracic surgery, 26 (28.9%) underwent abdominal surgery, and 17 (18.9%) underwent surgery involving an extremity. Among the patients undergoing brain surgery, the proportion of those in whom epinephrine was undetectable in serum was 89% in those who received some combination of norepinephrine and vasopressin, compared with 60% in those who did not. Among the patients undergoing thoracic surgery, those proportions were 77% and 47%, respectively.

**Table 5:** Patients receiving vasopressin, norepinephrine, or both, by age group and detectability of serum epinephrine

Age group	Serum epinephrine		Total	*p*-value*	
	Undetectable	Detectable			OR (95% CI)
	*n* (%)	*n* (%)	*n* (%)		
≤ 60 years					
Vasopressin, norepinephrine, or both					
No	12 (28.6)	5 (11.9)	17 (40.5)	1.00	1.00 (Reference)
Yes	17 (40.5)	8 (19.0)	25 (59.5)		0.88 (0.23; 3.38)
Total	29 (69.1)	13 (30.9)	42 (100.0)		
> 60 years					
Vasopressin, norepinephrine, or both					
No	11 (27.5)	7 (17.5)	18 (45.0)	0.05	1.00 (Reference)
Yes	20 (50.0)	2 (5.0)	22 (55.0)		6.36 (1.12; 36.08)
Total	31 (77.5)	9 (22.5)	40 (100.0)		

Patients who were receiving epinephrine (*n* = 8) were excluded from the analysis

*OR* odds ratio; *95% CI* 95% confidence interval

*Fisher’s exact test

### In-hospital mortality and serum levels of norepinephrine

We also stratified the patients by norepinephrine level—≤ 2006.5 pg/mL vs. > 2006.5 pg/mL and adjusted by detectability of epinephrine (Table 6). For the patients with an undetectable level of epinephrine, the in-hospital mortality was higher among patients with a norepinephrine level ≥ 2006.5 pg/mL (OR [95% CI] = 4.00 [1.27, 12.58]), *p* = 0.03). We found no evidence to suggest that the serum level of norepinephrine was associated with in-hospital mortality when the serum epinephrine level is detectable (Table 6).

**Table 6:** Frequency distribution of in-hospital mortality and serum levels of norepinephrine, adjusted by detectability of epinephrine

	In-hospital mortality		Total	*p*-value*	
	No	Yes			OR (95% CI)
	*n* (%)	*n* (%)	*n* (%)		
Undetectable					
Serum norepinephrine ≥ 2006.5 pg/mL					
No	24 (40.0)	6 (10.0)	30 (50.0)	0.03	1.00 (Reference)
Yes	15 (25.0)	15 (25.0)	30 (50.0)		4.00 (1.27; 12.58)
Total	39 (65.0)	21 (35.0)	60 (100.0)		
Detectable					
Serum norepinephrine ≥ 2006.5 pg/mL					
No	7 (23.3)	2 (6.7)	9 (30.0)	0.68	1.00 (Reference)
Yes	14 (46.7)	7 (23.3)	21 (70.0)		1.75 (0.29; 10.74)
Total	21 (70.0)	9 (30.0)	30 (100.0)		

*OR* odds ratio; *95% CI* 95% confidence interval

*Fisher’s exact test

### In-hospital mortality

The logistic regression analyses for age, gender, SOFA score, SAPS III, Glasgow coma score, mechanical ventilation, vasoactive drugs, detectability of epinephrine, levels of norepinephrine, and lactate levels are presented in Table 7. The SOFA score, SAPS III, Glasgow score, mechanical ventilation and vasoactive drugs were associated with in-hospital mortality (Table 7). In the > 60-year age group, the mortality rate was 47.6%, compared with 20.8% in the ≤ 60-year age group (OR [95% CI] = 3.45 [1.37, 8.70], *p* = 0.01). Mortality was higher among patients with norepinephrine levels above 2006.5 pg/mL (OR [95% CI] = 2.94 [1.13, 7.64], *p* = 0.02).

**Table 7:** Frequency distribution of in-hospital mortality adjusted by age, gender, SOFA score, SAPS III, detectability of epinephrine, levels of epinephrine, mechanical ventilation and use of vasoactive drugs

		In-hospital mortality		All	*p*-value*	OR (95% CI)
		Yes	No			
		*n* (%)	*n* (%)			
Age						
	≤ 60	10 (20.8)	38 (79.2)	48 (53.3)	**0.01**	1.00 (Reference)
	> 60	20 (47.6)	22 (52.4)	42 (46.7)		3.45 (**1.37; 8.70**)
Gender						
	Male	17 (27.4)	45 (72.6)	62 (68.9)	0.09	1.00 (Reference)
	Female	13 (46.4)	15 (53.6)	28 (31.1)		2.29 (0.91; 5.81)
SOFA score						
	1 (< 9)	12 (21.4)	44 (78.6)	56 (62.2)	< 0.01	1.00 (Reference)
	2 (9–11)	9 (52.9)	8 (47.1)	17 (18.9)		4.13 (1.31; 12.98)
	3 (> 12)	9 (52.9)	8 (47.1)	17 (18.9)		4.13 (1.31; 12.98)
Epinephrine						
	1 (Detectable)	9 (30.0)	21 (70.0)	30 (33.3)	0.81	1.00 (Reference)
	0 (Undetectable)	21 (35.0)	39 (65.0)	60 (66.7)		1.26 (0.49; 3.23)
Norepinephrine						
	1 (< 2006.5)	8 (20.5)	31 (79.5)	39 (43.3)	0.02	1.00 (Reference)
	2 (≥ 2006.5)	22 (43.1)	29 (56.9)	51 (56.7)		2.94 (1.13; 7.64)
Glasgow						
	1 (3–8)	22 (43.1)	29 (56.9)	51 (56.7)	< 0.01	5.12 (1.56; 16.78)
	2 (9–12)	4 (50.0)	4 (50.0)	8 (8.9)		6.75 (1.19; 38.40)
	3 (13–15)	4 (12.9)	27 (87.1)	31 (34.4)		1.00 (Reference)
SAPS III						
	1 (17–41)	2 (9.1)	20 (90.9)	22 (24.5)	< 0.01	1.00 Reference
	2 (42–52)	4 (19.0)	17 (80.9)	21 (23.3)		2.35 (0.38; 14.47)
	3 (53–63)	10 (47.6)	11 (52.4)	21 (23.3)		9.09 (1.68; 49.12)
	4 (64–103)	14 (53.8)	12 (46.2)	26 (28.9)		11.67 (2.25; 60.47)
Mechanical Ventilation						
	0 (yes)	26 (43.3)	34 (56.7)	60 (66.7)	< 0.01	4.97 (1.54; 16.01)
	1 (no)	4 (13.3)	26 (86.7)	30 (33.3)		1.00 Reference
Vasoactive drugs						
	0 (yes)	24 (80.0)	31 (51.7)	55 (61.1)	0.01	1.00 Reference
	1 (no)	6 (20.0)	29 (48.3)	35 (38.9)		3.74 (1.34; 10.46)
Lactate						
	1 (normal)	22 (75.9)	49 (84.5)	71 (81.6)	0.23	1.00 Reference
	2 (moderately elevated)	2 (6.9)	6 (10.3)	8 (9.2)		1.35 (0.25; 7.21)
	3 (severely elevated)	5 (17.2)	3 (5.2)	8 (9.2)		0.27 (0.06; 1.23)

*OR* odds ratio; *95% CI* 95% confidence interval

*Fisher’s exact test

## Discussion

We have demonstrated that the use of vasopressin or norepinephrine is associated with serum epinephrine undetectability in patients over 60 years of age. We have also demonstrated that, when serum levels of epinephrine is undetectable, a serum norepinephrine level ≥ 2006.5 pg/mL is associated with a greater risk of death. Therefore, we speculate that patients with a lower intrinsic sympathetic response would be at a higher risk for poor outcomes and require more vasopressors in general.

In trauma patients, hemodynamic stability is achieved through adequate volume resuscitation and the use of vasoactive drugs. Various types of stimuli (trauma, hypovolemia, pain, anxiety, inflammation, stress, and infections) increase activity in the sympathetic nervous system, resulting in epinephrine release by the adrenal medulla as an adaptive response [9–11]. Emergency surgery, whether in the setting of trauma or not, increases sympathetic nervous system activity.

Catecholamines are among the first mediators to be released in a stress response. Once released into the blood, these stress hormones exert multiple effects, especially on the cardiovascular system, leading to appropriate adjustments of blood pressure and heart rate, as well as of the energy metabolism, enabling the organism to respond to threats to its survival [12]. To ensure appropriate catecholamine secretion, the needs of which differ considerably from basal to stressful conditions, there is continuous adaptation of the stimulus-secretion coupling of chromaffin cells in the adrenal medulla. That adaptation occurs through transient or persistent remodeling of crucial molecular, cellular, and tissue elements of the medulla [12]. The mechanisms of such adaptation include neurotransmission between the splanchnic nerve and chromaffin cells at the cholinergic splanchnic–adrenal synapse, intercellular communication among chromaffin cells at the intercellular gap junction, and activation of voltage-gated calcium channels on the chromaffin cell membranes [12, 13]. A number of situations commonly seen in critically ill patients are related to the poor functioning of one or more of those mechanisms, such as the hypothalamic–pituitary–adrenal axis dysfunction observed in head trauma patients [14–16]; cellular-level damage to the adrenal gland due to hypoxia or to local or systemic pH changes; and a progressive decline in epinephrine release due to prolonged circulatory shock, which leads to chromaffin cell depletion [17].

It is estimated that dysfunction of the hypothalamic–pituitary–adrenal axis occurs in up to 20% of critically ill patients and in 60% of those with sepsis, abolishing or reducing the intensity of cortisol activity [18, 19]. The reported incidence of such dysfunction is higher in patients over 60 years of age [20, 21], which is in keeping with our findings in the present study. The higher odds ratio for epinephrine undetectability in our > 60-year age group also corroborates the established concept of the frequency of hypothalamic–pituitary–adrenal axis dysfunction being higher in critically ill patients over 60 years of age, as described by Rushworth et al. [20, 22]. Similarly, Johansson et al. [6] reported that epinephrine levels were lower in older trauma patients.

By measuring the variability in heart rate and the sensitivity of chemoreceptors and baroreceptors, Schmidt et al. [21] demonstrated a link between attenuation of the autonomic response and higher mortality in critically ill patients. We hypothesized that the undetectability of epinephrine would be due to attenuation of the sympathetic response. Abboud et al. [23] published data suggesting that exogenous epinephrine alters the endogenous norepinephrine metabolism in patients with sepsis. However, that phenomenon has not been described in trauma patients.

Our study has some limitations. First, it was an observational study, with no control group. In addition, we did not have access to the data collected before ICU admission and before the administration of vasoactive drugs, which could have helped us better analyze the autonomic mechanism involved. Another limitation was the heterogeneity of the sample, in terms of the severity of the trauma and the inclusion of non-trauma patients. Furthermore, the fact that we used single rather than serial measurements made it difficult to estimate the intensity and duration of the hypothalamic–pituitary–adrenal axis dysfunction and reduced the accuracy of its correlation with the variables evaluated. Further studies with larger patient samples are needed in order to corroborate our findings. Nonetheless, our study raises an important question: In older critically ill patients, should we start administering higher doses of epinephrine when serum norepinephrine levels are already high? The use of epinephrine might improve the clinical prognosis in older surgical patients with shock.

In conclusion, there is an evident association between age and mortality in surgical critical care patients. An undetectable level of epinephrine, which appears to be more common in older patients, could contribute to poor outcomes in such patients.

## Data Availability

No datasets were generated or analysed during the current study.
